# A phenome-wide scan reveals convergence of common and rare variant associations

**DOI:** 10.1186/s13073-023-01253-9

**Published:** 2023-11-28

**Authors:** Dan Zhou, Yuan Zhou, Yue Xu, Ran Meng, Eric R. Gamazon

**Affiliations:** 1 School of Public Health and the Second Affiliated Hospital, Zhejiang University School of Medicine, Hangzhou, China; 2 Division of Genetic Medicine, Department of Medicine, Vanderbilt University Medical Center, Nashville, TN USA; 3 Vanderbilt Genetics Institute, Vanderbilt University Medical Center, Nashville, TN USA; 4 The Key Laboratory of Intelligent Preventive Medicine of Zhejiang Province, Hangzhou, China; 5 Department of Biostatistics and Center for Quantitative Sciences, Vanderbilt University Medical Center, Nashville, TN USA; 6 Data Science Institute, Vanderbilt University Medical Center, Nashville, TN USA

**Keywords:** Genome-wide association study, Complex trait, Genetic architecture, Common and rare variants

## Abstract

**Background:**

Common and rare variants contribute to the etiology of complex traits. However, the extent to which the phenotypic effects of common and rare variants involve shared molecular mediators remains poorly understood. The question is essential to the basic and translational goals of the science of genomics, with critical basic-science, methodological, and clinical consequences.

**Methods:**

Leveraging the latest release of whole-exome sequencing (WES, for rare variants) and genome-wide association study (GWAS, for common variants) data from the UK Biobank, we developed a metric, the COmmon variant and RAre variant Convergence (CORAC) signature, to quantify the convergence for a broad range of complex traits. We characterized the relationship between CORAC and effective sample size across phenome-wide association studies.

**Results:**

We found that the signature is positively correlated with effective sample size (Spearman *ρ* = 0.594, *P* < 2.2e − 16), indicating increased functional convergence of trait-associated genetic variation, across the allele frequency spectrum, with increased power. Sensitivity analyses, including accounting for heteroskedasticity and varying the number of detected association signals, further strengthened the validity of the finding. In addition, consistent with empirical data, extensive simulations showed that negative selection, in line with enhancing polygenicity, has a dampening effect on the convergence signature. Methodologically, leveraging the convergence leads to enhanced association analysis.

**Conclusions:**

The presented framework for the convergence signature has important implications for fine-mapping strategies and drug discovery efforts. In addition, our study provides a blueprint for the expectation from future large-scale whole-genome sequencing (WGS)/WES and sheds methodological light on post-GWAS studies.

**Supplementary Information:**

The online version contains supplementary material available at 10.1186/s13073-023-01253-9.

## Background

The gap between chip-based heritability and the narrow-sense heritability estimated from twin studies suggests a substantial role for rare variants in the etiology of complex traits [1–3]. Empirically, it has been observed that up to approximately 22% of the phenotypic variance can be explained by rare variants [4]. Since rare variants have historically been excluded in genome-wide scans, the contribution of this class of variants to complex traits has been much less understood [1, 5, 6]. Several studies have suggested that common and rare variants may play distinct etiological roles [7]. Mediated by quantitative molecular traits, the variance of common variants constitutes the background of disease liability according to the infinitesimal model, while most deleterious rare variants modify the liability through protein dysfunction [8, 9].

Although some studies appear to show that the signals from whole-exome sequencing (WES) diverge in function from those from genome-wide association studies (GWAS) [10–13], these studies are limited in their effective sample size. By contrast, recent studies with relatively large sample sizes report that most rare variants implicate loci which have been previously identified by common variants [14–16], indicating some level of convergence on mediating genes. A more recent study shows that common and rare variants partially colocalize at individual genes and loci across 22 complex traits [17]. The extent of this convergence is a fundamental question in human genetics that remains poorly understood. Methodologically, it may enable the development of a rigorous strategy to fine-map a genomic region of interest, allowing discrimination of causal mechanisms.

Given recent results from some relatively well-powered empirical studies, we hypothesized the presence of a substantial degree of functional convergence after accounting for sample size and heritability. The increasing availability of common variant and rare variant genomic datasets provides an opportunity to gain new insights into the genetic architecture of complex traits by extrapolating the degree of concordance. Leveraging common variant-based GWAS and rare variant-based WES [18] and a broad collection of phenotypes with a wide range of effective sample sizes [12, 18] from a large-scale biobank, we set out to investigate the concordance of common and rare genetic influences on complex traits, using a newly defined Common variant and Rare variant Convergence (CORAC) signature. We examined a potential mechanism on the phenome underlying the patterns of shared or divergent mediation.

## Methods

### Common variant analysis

The GWAS summary statistics for common variants were obtained from the Neale Lab (http://www.nealelab.is/uk-biobank) [19]. After quality control, variants with minor allele frequency (MAF) ≥ 0.05 were included. Up to 337,199 subjects were available, with the effective sample size varying with the trait. The genome-wide association statistics had been estimated using a linear model with sex and the first 10 principal components as covariates. We mapped the common variants to protein-coding genes. The generalized gene-set analysis approach MAGMA [20] with default settings was implemented to project the SNP-level signal to a gene level signal (*P*_*common*_). The common variant-based heritability was estimated using *ldsc* applied to the GWAS summary statistics.

### Rare variant analysis

The rare variant analysis was performed for a set of phenotypes in the UK Biobank dataset of 450,953 individuals. A total of 263,696 rare variants were annotated using VEP v95, as implemented in Hail with default parameters, and grouped into three annotation categories, including pLoF (high-confidence by LOFTEE), missense|LC (missense variants and variants annotated as low-confidence by LOFTEE), and synonymous. For each category, gene-based burden tests and SKAT-O tests were performed for each of the 19,407 protein-coding genes [21, 22]. The summary-level results were accessed from the Genebass portal through Hail (https://hail.is/) [18]. For each gene, the minimum *p*-value (*P*_*rare*_) among the six tests (two statistical tests × three annotation categories) was used in downstream analyses.

### Estimation of level of convergence on shared effector genes

To estimate the convergence of common and rare variants on shared molecular mediators, variant-level signals were mapped to gene-level signals as described above. For each approximately independent linkage disequilibrium (LD) block [23], *p*-value-based (the product of *P*_*common*_ and *P*_*rare*_) clumping was performed to reduce the complexity of the LD structure. i.e., for each LD block, one gene with the highest association signal was included.

To control for the effect of sample size on the number of significant genes, the top-ranked 100 genes were selected from both common and rare variant-based gene lists as the significant genes. Sensitivity analysis was performed using top 20, top 50, and top 200 genes. Let *a*, *b*, *c*, and *d* denote the number of genes showing significance from both the common and rare variant-based tests, only from the common variant-based test, only from the rare variant-based test, and neither from the rare nor common variant-based test, respectively. Let $n=a+b+c+d$ be the total number of genes. Let ${p}_{11}$, ${p}_{10}$, ${p}_{01}$, and ${p}_{00}$ denote the corresponding probabilities for the 4 sets of genes, respectively.

We define the Common variant and Rare variant Convergence (CORAC) signature, which quantifies the concordance of implicated genes for common and rare variants. The proportionate agreement (${p}_{\mathrm{\alpha }}={p}_{11}+{p}_{00}$) is estimated using the gene-count statistics:

$$\widehat{{p}_\mathrm{\alpha}}=\frac{a+d}n$$

The expected probability that both common and rare variants show a high-ranking signal at random (${p}_{\mathrm{\beta} }={p}_{1.} \times {p}_{.1}$) is estimated as follows:

$$\widehat{{p}_{\mathrm{\beta} }}=\frac{a+b}n\times\frac{a+c}n$$

The expected probability that a gene implicated by neither common nor rare variants shows a high-ranking signal at random (${p}_{\mathrm{\delta} }={p}_{0.} \times {p}_{.0}$) is estimated as follows:

$${\widehat{{p}_{\mathrm{\delta}}}}=\frac{c+d}{n}\times \frac{b+d}{n}$$

The overall random agreement probability ${p}_{\mathrm{\epsilon}}$ is the sum of ${p}_{\mathrm{\beta} }$ and ${p}_{\mathrm{\delta} }$. CORAC is given by the Cohen’s kappa coefficient $\kappa$:

1 $$\kappa =\frac{{p}_{\mathrm{\alpha}}-{p}_{\mathrm{\epsilon}}}{1-{p}_{\mathrm{\epsilon}}}$$

Note the estimator $\widehat{\kappa }$ is a chance-corrected statistic (via ${\widehat{{p}_{\mathrm{\epsilon}}}}$). As a measure of agreement, $\kappa$ is to be contrasted with the Fisher’s exact test and the ${\chi }^{2}$ test, which assign the same *p*-value to perfect agreement or perfect disagreement. Furthermore, odds ratio has a problematic scale; it equals 1 in the case of random agreement and infinity in the absence of error, rendering comparison difficult to interpret.

To quantify the standard error of the estimator and facilitate downstream statistical inference, we performed bootstrap. Alternatively, one can conduct posterior inference from a Bayesian model [24] to quantify the uncertainty. The likelihood is given by:

$$\mathcal{L}=\frac{n!}{a!b!c!d!}[{p}_{11}]^{a}{[{p}_{10}]}^{b}{[{p}_{01}]}^{c}{[{p}_{00}]}^{d}=\frac{n!}{a!b!c!d!}[{p}_{11}]^{a}{[{p}_{1.}-{p}_{11}]}^{b}{[{p}_{.1}-{p}_{11}]}^{c}{[1-{p}_{1.}-{p}_{.1}-{p}_{11}]}^{d}$$

Note this likelihood is a function of ${p}_{1.}$, ${p}_{.1}$, and ${p}_{11}$. The prior on ${p}_{1.}$ And ${p}_{.1}$ can be assumed to be:

$${p}_{1.} \sim \mathrm{ Beta}({u}s,{u}\left(1-s\right))$$

$${p}_{.1} \sim \mathrm{ Beta}({v}t,{v}\left(1-t\right))$$

where $0<s,t<1$, respectively. The prior on ${p}_{11}$ is a uniform distribution. Using the likelihood and the choice of prior, the posterior distribution can then be used to obtain the posterior mean and the credible interval.

The Spearman’s correlation coefficient *ρ* was then calculated between the CORAC estimate $\widehat{\kappa }$ and the effective sample size.

In addition, we define a modified statistic CORAC_modified_, which has some methodological advantages. CORAC_modified_ is less dependent on the prevalence. i.e., the true proportion of associated genes, which may need to be considered in interpreting the agreement rate, allowing comparisons among phenotypes. CORAC_modified_ is given by Gwet’s AC1:

$$g=\frac{{p}_{\mathrm{\alpha} }-{p}_{\mathrm{\gamma} }}{1-{p}_{\mathrm{\gamma} }}$$

where

$${p}_{\mathrm{\gamma} }=2\pi \left(1-\pi \right)\mathrm{\ with\ }\pi =\frac{1}{2}{p}_{\mathrm{\beta} }=\frac{1}{2}({p}_{1.}+{p}_{.1})$$

The difference between the two convergence coefficients stems from how the adjustment for *chance* agreement between the common and rare variant signals is implemented (${p}_{\mathrm{\epsilon}}$ in $\kappa$ versus ${p}_{\mathrm{\gamma} }$ in $g$).

### Stratified analysis

For a given phenotype, we define statistics that quantify the extent to which rare (common, respectively) variant informed analysis improves our ability to detect genes from the common (rare, respectively) variant analysis. Following the stratified FDR [25] approach for GWAS, we calculated the posterior probability that a gene is null for the rare variants given that the associations from the rare variants and the common variants are at least as significant as the observed associations:

2 $$\mathrm{FDR}\left({p}_{\mathrm{R}}|{p}_{\mathrm{C}}\right)=\frac{{\pi }_{0}({p}_{\mathrm{C}}){p}_{\mathrm{R}}}{\mathrm{F}({p}_{\mathrm{R}}|{p}_{\mathrm{C}})}$$

Here, ${p}_{\mathrm{R}}$ is the *p*-value of the gene from the rare variant analysis, ${p}_{\mathrm{C}}$ is the corresponding *p*-value from the common variant analysis, ${\pi }_{0}({p}_{\mathrm{C}})$ is the conditional proportion of null genes for the rare variant analysis given that the *p*-values for the common variant analysis are as small as ${p}_{\mathrm{C}}$, and $\mathrm{F}({p}_{\mathrm{R}}|{p}_{\mathrm{C}})$ is the conditional cumulative distribution function. Similarly, we define the posterior probability $\mathrm{FDR}\left({p}_{\mathrm{C}}|{p}_{\mathrm{R}}\right)$ with ${p}_{\mathrm{R}}$ and ${p}_{\mathrm{C}}$ switched in Eq. (2).

This analysis was illustrated using a stratified Q-Q plot. This plot can be used to visualize the degree to which the use of gene-level associations from the rare (common, respectively) variant analysis enhances our ability to detect gene-level associations from the common (rare, respectively) variant analysis. Differential departure from the null across different *p*-value inclusion threshold criteria quantifies the enrichment due to the prior information independently of the presence of shared subjects in the common and rare variant analyses.

### Role of negative selection in convergence

We tested the extent to which negative selection impacts the functional convergence of common and rare variant associations. Negative selection has been proposed as a mechanism for the extreme polygenicity of complex traits characterized by the flattening of heritability across the genome [26]. Negative selection may also induce variant effect size to vary with linkage disequilibrium [27].

We considered a class of genetic architectures consistent with signatures of negative selection [28], i.e., where the allele frequency influences the allelic substitution effect at a causal variant as follows:

3 $${\beta }_{i} | \left({p}_{i} , {l}_{i}\right) \sim \mathcal{N}(0, \mathrm{C}{\left[{p}_{i}\left(1-{p}_{i}\right)\right]}^{1+\alpha }{\left\{{}^{1}\!\left/ \!{}_{\left(1+{l}_{i}\right)}\right.\right\}}^{r})$$

Here, the constant of proportionality $\mathrm{C}=\frac{{h}_{\mathrm{trait}}^{2}}{{N}_{\mathrm{trait}}}$ is given by the heritability ${h}_{\mathrm{trait}}^{2}$ divided by the number of causal variants ${N}_{\mathrm{trait}}$ and independent of the variant; ${p}_{i}$ is the allele frequency of the variant $i$; ${l}_{i}$ is the LD score; and $\alpha$ is a signature of selection on the trait linking the allele frequency of $i$ to the variance of SNP effects. We assume $r$ is either 0 (which corresponds to a MAF-dependent distribution of effect sizes) or 1 (which corresponds to a MAF- and LD- dependent distribution of effect sizes). The parameters ${p}_{i}$ and ${l}_{i}$ can be estimated from an ancestry-matched reference panel. In our framework, the LD score is integrated in the effect size distribution (Eq. 3), rather than downstream in the definition of CORAC, as one model of genetic architecture, through which LD influences the convergence signature. We assume that the genotype is scaled with mean 0 and variance 1. The model (Eq. 3) has been shown to be consistent with what is observed in the UK Biobank, with $\widehat{\alpha }=-0.37$ [29]. We note that the constant factor $\mathrm{C}$ is the expected value of the per-SNP heritability under a neutral model ($\alpha =0$), in which the causal effect size distribution is independent of allele frequency. An estimate of $\alpha$ can be obtained by maximizing the profile likelihood [30].

Here, we estimated $\widehat{\alpha }$ using the approximate joint log likelihood $\mathrm{log}{l}_{\mathrm{SS}}$ that can be calculated from summary statistics [31]. We computed the partial correlation between the convergence level $\widehat{\kappa }$ (Eq. 1) and $\widehat{\alpha }$ (Eq. 3) while adjusting for the effective sample size in addition to the Spearman correlation (without the adjustment).

We tested the robustness of the observation concerning the effect of negative selection on the degree of convergence by using another approach to detect the selection signal. We applied a Bayesian mixed model approach [29, 32] to infer the action of natural selection on the genetic variants underlying a phenotype. The approach estimates a parameter $S$ (with an asymptotic normal approximation to its posterior distribution) representing the relationship between the variance of SNP effects and minor allele frequency using genome-wide SNP data. We then tested the correlation of the estimate $\widehat{S}$ with the CORAC estimate $\widehat{\kappa }$.

### Simulation framework

We studied the behavior of the convergence level with respect to effective sample size in simulations. Towards this end, using actual (scaled) genotype data, we simulated genetic architectures consistent with negative selection (Eq. 3, with $r=0$) for comparison with a baseline class of genetic architectures (in which there is no dependence of the distribution of variant causal effect on allele frequency):

4 $${\beta}_{i} |\left({p}_{i},{l}_{i}\right)\sim \mathcal{N}\left(0,\frac{h_{\mathrm{trait}}^2}{{N}_{\mathrm{trait}}}\right)$$

We set the following parameters: heritability (0.30), the proportion of causal genes (10%), and the probability ${p}_{\mathrm{\alpha} }$ (0.50), i.e., the proportion of shared causal genes between common and rare variant-based signals. We varied the effective sample size (from 1000 to 10,000). For each class of genetic architectures, we generated $n$ simulations (100) with different seeds for sampling. We generated the phenotype and identified the gene-level signals from the common and rare variants. We investigated the Spearman correlation between $\kappa$ and sample size for each class of genetic architectures.

To examine the behavior of the correlation before and after decorrelating common and rare variants, we fixed the genotype for common variants and shuffled the genotype for each rare variant across individuals to break any potential correlation. Furthermore, we calculated the convergence level under different degrees of polygenicity by varying the proportion of causal genes across the genome (from 5 to 20%).

### Verification using independent data sources for common and rare variants

We further estimated the correlation between the convergence level and effective sample size using independent data sources for the common and rare variant-based signals. This analysis enabled us to evaluate the impact of the use of a shared biobank dataset for the common and rare variant analyses. We used the UK Biobank-free GWAS results from the GWAS ATLAS [33] as the source for the common variant-based associations. The GWAS results generated from any UK Biobank samples were excluded. The WES-based results from the UK Biobank were used as the source for the rare variant-based signals. Only European ancestry samples were included. To harmonize the phenotype data between the GWAS ATLAS and the UK Biobank, the Sentence-BERT [34] word embedding model was implemented [34]. The resulting embeddings from the Transformer-based network were used to search for semantic similarity. Phenotype pairs with cosine similarity less than or equal to 0.75 were filtered out. We then manually confirmed the resulting phenotype pairs and removed duplicated ones. The correlation between CORAC and effective sample size was estimated in this dataset.

## Results

### Gene signals from common and rare variants

Gene signals from common variants were estimated in 337,199 individuals from the UK Biobank. The generalized gene-set analysis approach MAGMA was implemented to map the SNP-level GWAS signals to gene-level signals. Leveraging the WES data from 450,953 UK Biobank participants, the burden test and SKAT-O test were applied to identify trait-associated genes by pooling rare variants. For each gene, the minimal *p*-value among the two tests across three annotation categories (pLoF, missense|LC, and synonymous) was used in the subsequent analyses. We did not adjust for gene length since the correlation between the significance of a common or rare variant-based gene and gene length was negligible (with median Spearman correlation coefficient < 0.02 across the traits). In total, 1043 traits were analyzed, having both common and rare variant-based genome-wide results available. We included 412 heritable traits with nominally significant *p*-value (*P* < 0.05) from the common variant-based *h*^*2*^ estimation (as implemented in ldsc) in the downstream analyses. These traits include body measurements, lab measurements, self-reported disorders, doctors’ diagnoses, and treatments. The effective sample size ranged from 596 to 394,432.

### Convergence signature

**Fig. 1:**
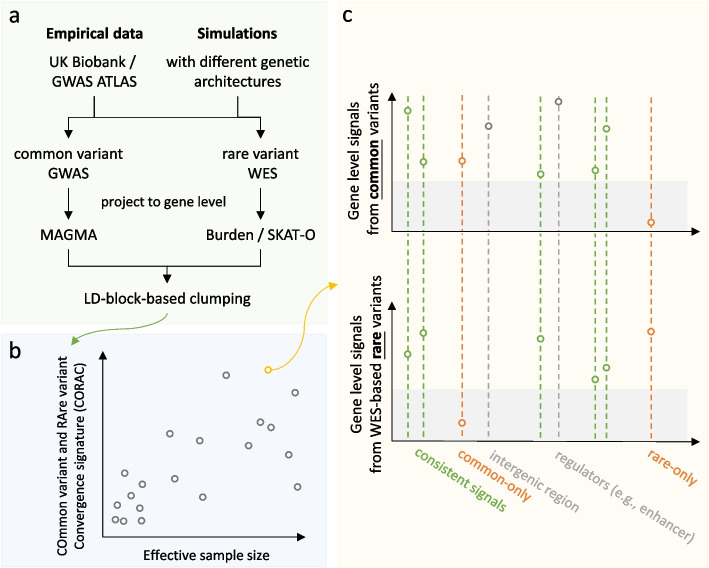
Study design. **a** Leveraging the empirical data (e.g., UK Biobank/GWAS ATLAS) and simulations of different genetic architectures, the signals from common and rare variants were projected to genes via MAGMA and Burden/SKAT-O, respectively. **b** Non-parametric and parametric tests were used to estimate the correlation between the effective sample size and the Common variant and Rare variant Convergence (CORAC) signature and an alternative chance-corrected convergence coefficient (CORAC_modified_). Sensitivity analysis was performed by varying the number of top-ranked significant genes. The standard error of the convergence coefficient was estimated using bootstrap, enabling downstream statistical inference. Posterior inference can be performed on the signature using a Bayesian framework (Methods). **c** Visualization of the convergence signature

An overview of our framework is shown in Fig. 1a, b, and c. CORAC was estimated for each trait, quantifying the rate of functional convergence from common variants and rare variants (Methods). Bootstrap was used to estimate the 95% confidence interval of the estimator (Additional file 2: Table S1.). The bootstrap distribution of CORAC can be viewed as its nonparametric posterior distribution under a non-informative prior within a Bayesian formulation (Methods).

**Fig. 2:**
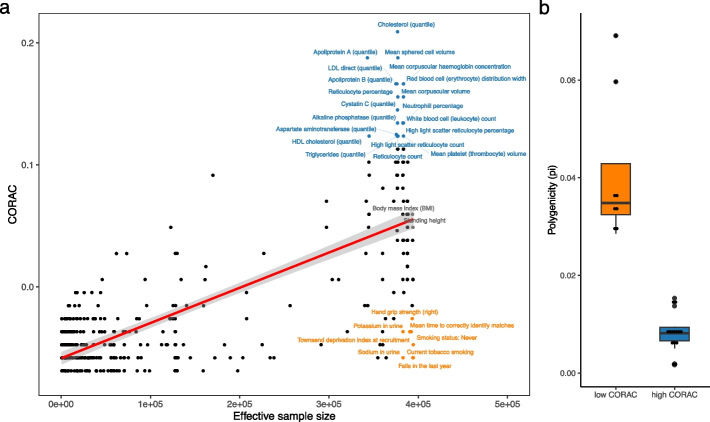
Convergence of common and rare genetic effects as a function of sample size. The CORAC statistic (*y*-axis) is positively correlated with effective sample size (*x*-axis). The positive relationship remained after accounting for heteroskedasticity (*P* < 2.2e − 16). The significant relationship was also observed after further adjustment for the number of detected association signals and after sensitivity analysis that varied the number of significant genes (20, 50, and 200 top genes). The regression line and the 95% confidence bands are shown, allowing identification of traits with a higher or lower convergence signature than expected given the effective sample size. Traits with relatively high and low CORAC values are colored blue and orange, respectively. Two traits (body mass index and standing height) located close to the regression line are colored grey (**a**). We implemented SbayesS and estimated the degree of polygenicity (pi) for each trait. Traits (colored orange in **a**) with low CORAC values show a significantly higher degree of polygenicity than traits (colored blue in **a**) with high CORAC values (*P* = 3.5e − 4, **b**)

The convergence level was found to be positively correlated with the effective sample size (Spearman *ρ* = 0.594, *P* < 2.2e − 16; Fig. 2a). A positive correlation was also observed between CORAC and the common-variant based heritability (Spearman *ρ* = 0.369, *P* = 9.0e − 15). The association was also estimated using a parametric test. Considering the heteroskedasticity, we applied generalized least squares estimation. In this case, the weight (model) was determined as the choice of the exponent which maximizes the value of the likelihood function. A higher CORAC level continued to be associated with a larger effective sample size (*P* < 2.2e − 16). Certain phenotype classes such as hematopoietic traits and biomarkers showed a relatively higher convergence level than the other trait classes. However, after accounting for sample size, the difference dropped substantially (Additional file 1: Fig. S1.). The use of odds ratio supported the significant correlation with sample size (Additional file 2: Table S1), but the statistic has a problematic scale for practical use as a measure of concordance (Methods).

The significance of this relationship remained when we further adjusted for the number of significant genes (MAGMA). Thus, the higher convergence level for a larger effective sample size was *not* due to the number of detected association signals. Indeed, for sensitivity analysis, we varied the number association signals by considering the top 20, top 50, and top 200 genes. The Spearman *ρ* with effective sample size ranged from 0.594 to 0.622 across the different cutoffs, indicating the robustness of our finding.

A modified statistic CORAC_modified_, which is defined to be less dependent on prevalence (i.e., the proportion of associated genes; Methods) and thus, through enhanced calibration, allows phenotypes to be compared, reinforces the significant association of the level of convergence with effective sample size (*P* < 2.2e − 16, Additional file 1: Fig. S2).

We identified phenotypes with an unexpectedly high CORAC coefficient. In Fig. 2a, phenotypes away from the red line are labeled. In the top right corner, a group of lab measurements showing a high degree of convergence is highlighted. The Manhattan plot for cholesterol is displayed in Fig. 5a. Among the 100 top-ranked genes identified by common variants, 26 were also highly ranked from the rare variant-based test.

### Polygenicity and the convergence signature

We implemented SbayesS [29, 32] and estimated the degree of polygenicity for each trait. We observed that traits (colored orange in Fig. 2a) with low convergence levels (CORAC) have a significantly higher degree of polygenicity than traits (colored blue in Fig. 2a) with high convergence levels (*P* = 3.5e − 4, two sample *t*-test, Fig. 2b). This comparison was conducted in traits with similar effective sample sizes (~ 400 k), thereby minimizing any potential confounding effect of this variable. We also calculated the Spearman correlation between the degree of polygenicity and the convergence level across all available traits (in Fig. 2a). We observed that a higher degree of polygenicity tends to show a lower convergence level (Spearman *ρ* = -0.310, *P* = 2.3e − 10). This result held robustly after accounting for effective sample size (Kendall’s *τ* coefficient = -0.266, *P* = 1.8e − 15).

### Simulations

**Fig. 3:**
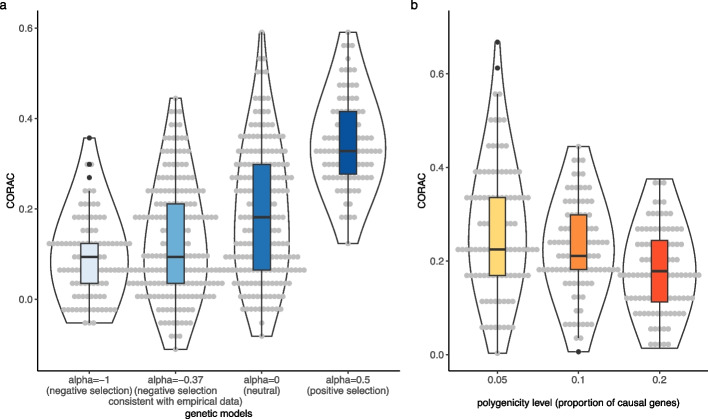
Simulations indicate that negative selection, in line with enhancing polygenicity, dampens the convergence level. Using empirical genotype data from the UK Biobank, we simulated the effect size for each causal variant by fixing the total *h*^2^ = 0.3 under genetic models consistent with negative selection (*α* =  − 1, *α* =  − 0.37), a neutral model (*α* = 0), and positive selection (*α* = 0.5). The CORAC coefficients observed for these models are shown in both violin plot and boxplot along with the data points in grey (**a**). We also varied the proportion of causal genes as a proxy for polygenicity and observed decreased CORAC with increased polygenicity (**b**)

Simulations were performed to evaluate CORAC under different genetic architectures (Methods). Briefly, using empirical genotype data from the UK Biobank, we varied the negative selection parameter α (from − 1 to 0.5) to investigate the convergence level under a range of scenarios: the neutral model (*α* = 0) and the model consistent with negative selection (*α* =  − 0.37, the average across complex traits estimated from the empirical data [29, 31]). In each case, we performed 100 simulations with different seeds for sampling. Compared with the neutral model (*α* = 0), the model consistent with negative selection (*α* =  − 0.37) tends to show a lower convergence level, a pattern supported by a clear dose–response trend (Fig. 3a). Furthermore, in simulations, we varied the proportion of causal genes across the genome (from 5 to 20%) as a proxy for the degree of polygenicity. A higher degree of polygenicity showed a lower convergence level (Fig. 3b), an observation consistent with our empirical results above. Altogether, our simulations demonstrate that traits under strong negative selection, in line with a high degree of polygenicity, would have a dampened level of convergence. This assertion is supported by both empirical data and simulations.

**Fig. 4:**
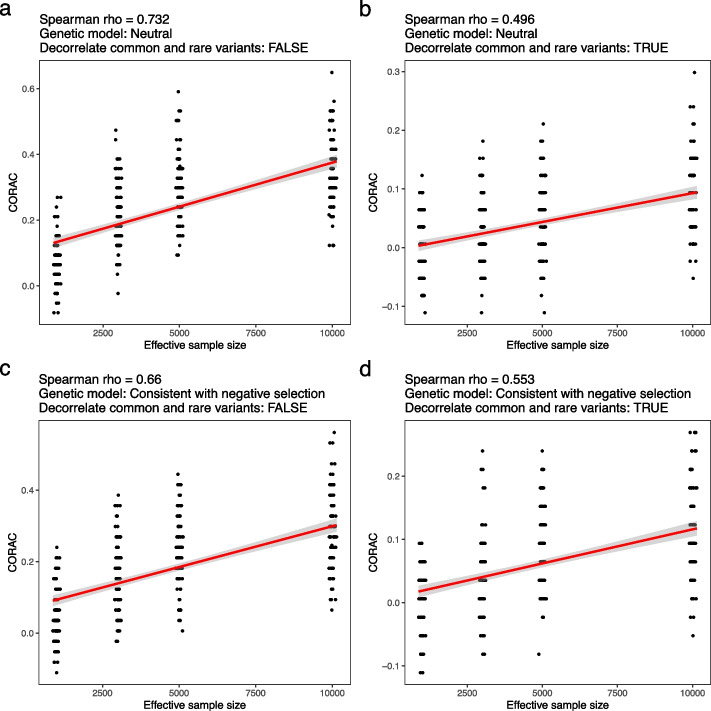
Simulations reveal the relationship between CORAC and effective sample size under different genetic architectures. Using empirical genotype data from the UK Biobank, we simulated the effect size for each causal variant by fixing the total *h*^2^ = 0.3 in genetic architectures consistent with a neutral model (*α* = 0, **a** and **b**) and consistent with negative selection (*α* =  − 0.37, **c** and **d**). We also investigated the impact of linkage disequilibrium on CORAC by decorrelating the common and rare variants (**b** and **d**; Methods). We varied the effective sample size (from 1000 to 10,000). The Spearman correlation coefficient between CORAC and effective sample size was calculated for each configuration

To determine the behavior of CORAC in the case of independent common and rare variants, we performed additional simulations that kept these two classes of variants (MAF cutoff: 0.01) independent. We fixed the genotype matrix (SNPs x individuals) for the common variants and shuffled the genotype matrix across individuals for each rare variant to decorrelate the two classes of variants. The CORAC coefficient decreased with decorrelated common and rare variants relative to the original (correlated) dataset (Fig. 4a and c). However, the positive correlation between CORAC and effective sample size continued to hold robustly (Fig. 4b and d). Indeed, among these scenarios, the model that assumes negative selection and decorrelated common and rare variants (Spearman *ρ* = 0.553) provides a reasonably good fit to the real data (Spearman *ρ* = 0.594).

### Enhanced association analysis by utilizing the convergence

**Fig. 5:**
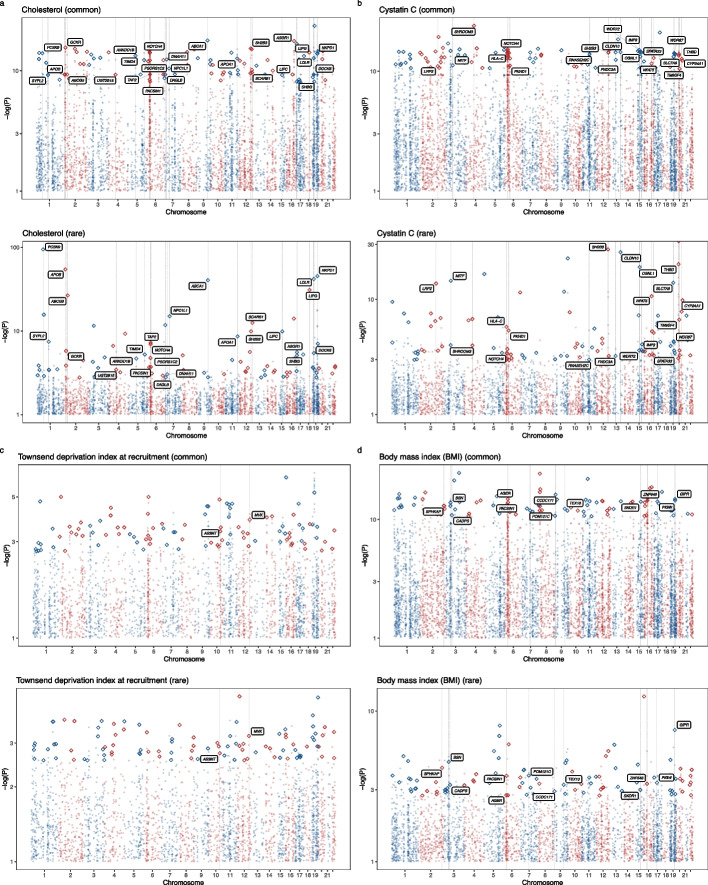
Comparisons of gene-level signals from common and rare variants. The CORAC signature, a global statistic, and the locus-specific convergence from common and rare variants highlight the diversity of genetic architectures of human complex traits. The common and variant-based gene-level Manhattan plots illustrate the convergence level. The hollow diamonds denote the top 100 genes. Genes highly ranked in both common and rare-variant-based tests are labeled with the gene symbol and a dashed vertical line. Traits with high (**a** and **b**), low (**c**), and average (**d**) CORAC coefficients are visualized

The CORAC signature may be a useful guide in restricting the search space for trait-associated genes. Stratified analysis (Methods) showed that conditioning on the common variant gene-level results significantly improved identification of rare variant implicated genes (Additional file 1: Fig. S3a). The observed differential departure from the null across different conditioning *p*-value thresholds implies that this gain was not due to the presence of the shared samples from the two sets of analyses. For example, the pattern was seen for cholesterol and cystatin C with 26 and 20 genes ranked at the top in both common and rare variant analyses, respectively (Fig. 5a, b, Additional file 1: Fig. S3a, and Fig. S3b). On the other hand, the Townsend deprivation index at recruitment (bottom right corner) showed a different pattern. Although the sample size was large (*N* = 394,375), the limited heritability (*h*^*2*^ = 0.031, *P* = 3.70e − 37) resulted in fewer significant signals from both the common and rare variant analyses and limited level of convergence (Fig. 5c and Additional file 1: Fig. S3c). With a large sample size, BMI and height showed a reasonable number (12 and 11, respectively) of overlapped genes (Fig. 5d and Additional file 1: Fig. S3d). Surprisingly, only one gene was co-mapped by the common and rare variant analyses for current tobacco smoking, a heritable trait with a large sample size. The CORAC signature estimate for each trait can be found in Additional file 2: Table S1. These results on signal convergence illustrate the diversity of genetic architectures across human complex traits.

### Verification of the CORAC-sample size correlation in independent sources of common and rare variant-based signals

**Fig. 6:**
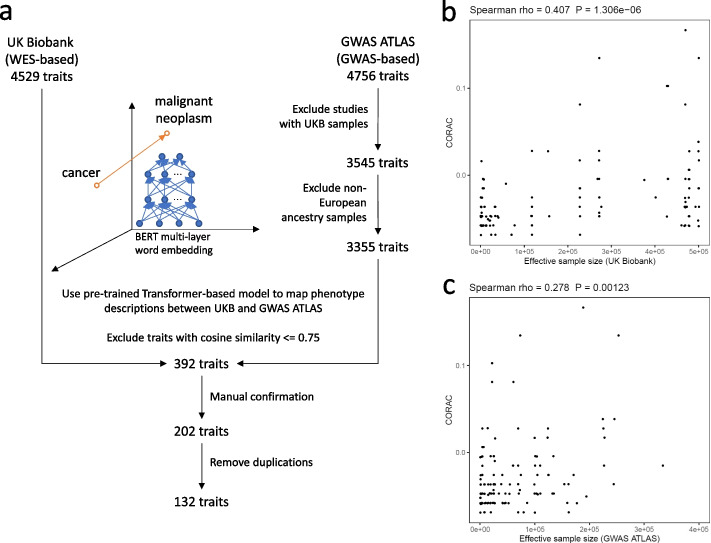
Independent data sources for the common and rare variant-based signals verified the correlation between CORAC and effective sample size. Instead of the UK Biobank as the data source for common variant-based signals, we leveraged the data from GWAS ATLAS to investigate the impact of the use of a shared dataset from which the common and rare variant signals were derived. Here, GWAS data that included any samples from the UK Biobank were excluded from the data source for the common variant-based signals. A pretrained Sentence-BERT word embedding model was implemented. The Transformer-based network searches for semantic similarity, enabling the mapping of phenotype descriptions in the UK Biobank to those in the GWAS ATLAS. Cosine similarity analysis (for semantic textual similarity), manual confirmation, and removal of duplications were then performed (**a**; Methods). The scatter plot shows the correlation between CORAC and effective sample size derived from the shared UK Biobank dataset (**b**) and from the independent data sources (**c**)

In the analyses above, both the common and rare variant-based signals were derived from the shared UK Biobank dataset, which could inflate the observed correlation between convergence level and effective sample size. We thus investigated this relationship using the UK Biobank-free results from GWAS ATLAS as the source for common variant-based signals (Fig. 6a). Here, GWAS results derived from any UK Biobank samples were excluded and only the European ancestry samples were included. To harmonize the phenotype data between the GWAS ATLAS and the UK Biobank, the pretrained Sentence-BERT word embedding model was implemented (Fig. 6a) to search for semantic similarity. Phenotype pairs with cosine similarity less than or equal to 0.75 were excluded, followed by manual confirmation and removal of remaining duplications. In all, 132 traits were matched (Additional file 2: Table S2). Both common variant and rare variant-based signals were estimated as before. Notably, we confirmed the positive correlation between the convergence level and the effective sample size, the latter either from the rare (Fig. 6b, Spearman *ρ* = 0.407, *P* = 1.31e − 6) or common (Fig. 6c, Spearman *ρ* = 0.278, *P* = 1.23e − 3) variant-based data source.

## Discussion

Limited by the scale of the WGS/WES studies to date, it was far from clear to what extent common and rare variants would colocalize and induce phenotypic effects through the same effector genes. Using variants across the entire MAF spectrum and a broad set of phenotypes with a range of sample sizes, the UK Biobank provides a great opportunity to investigate the patterns of shared or divergent mediation through effector genes. In general, our results show that the convergence from common and rare variants becomes even greater as the study sample size increases. The observation was confirmed when leveraging the UK Biobank-free GWAS results from the GWAS ATLAS [33] as the source for the common variant signals. Thus, future studies will be expected to identify a substantial proportion of shared gene mediators. Although the concordance may be partially explained by the synthetic associations of common variants with nearby rare variants, in general, common variants identified to date have not been found to be driven by synthetic associations [35].

Although empirical data indicate that rare variants are likely to produce phenotypic effects through protein-structure alteration while common variants are likely to act through regulation of gene expression, it is not contradictory for variants across the allele frequency spectrum to have shared effector genes. Our observations are consistent with the notion that trait-associated genes may mediate their effects across a phenotypic continuum through a mechanistic continuum, i.e., through lower expression level (likely common variant driven) or by loss of function due to a structural change (likely rare variant driven). Furthermore, rare variants do not always exert their phenotypic effects via protein-structure alteration. For example, low frequency xQTLs (where x may be gene expression, splicing, methylation, or another molecular trait) have been reported [36]. With convergence on the same effector genes, the challenge of fine-mapping causal genes, e.g., among gene-level associations derived from MAGMA, PrediXcan, and similar methods [37–39], could be informed by incorporating rare variant signals. Recently, Weiner and colleagues observed statistical and functional convergence of common and rare genetic influences on autism at chromosome 16p [40]. Interestingly, the 16p-specific polygenic risk score (PRS, representing the common variant burden) and the 16p11.2 CNV (representing a rare variant burden) resulted in a similar pattern of transcriptional effect for the genes on 16p, suggesting a potential shared mechanism [40]. Weiner and colleagues also showed that rare-variant heritability enrichment and common-variant enrichment were approximately equal for sets of genes specifically expressed in trait-matched cell and tissue types [17].

In our analyses, lab measurements ranked high in their level of convergence. These traits—for example, cholesterol traits, a class of well-studied traits with a large number of replicable loci—have a better (for example, less heterogeneous) quality of measurement, which may partially explain the observed high concordance. As representative polygenic traits, BMI and height showed a moderate level of convergence. However, the presence of some heritable traits (e.g., current smoking) showing a limited level of convergence is informative. The level of convergence for these traits raises the question of whether patterns of shared or divergent mechanisms are a critical feature or consequence of the architecture of complex traits.

Interestingly, we observed a lower convergence level for traits with a high degree of polygenicity even with large sample sizes. This finding is observed in empirical data, supported by simulations, and consistent with a previous report that extreme polygenicity of complex traits can be explained by negative selection [26]. Under negative selection, which purges large-effect mutations in critical regions and generates an architecture with a high degree of polygenicity from common variants, genes from the common variants are constrained to have modest effects and scattered throughout the genome whereas the causal rare variant associations have very large effects and are less diffused. Thus, the genes from the common variants and the genes from the rare variants would likely differ.

The consequences of these observations are substantial for study design and for our understanding of the joint effects of common and rare variants. First, fine-mapping of causal genes in GWAS or transcriptome-wide association study (TWAS)-implicated regions through rare variant data from sequencing studies could be more challenging under strong negative selection. Second, if estimates of $\alpha$ linking allele frequency to the variance of SNP effects lean towards more negative values, future WGS based GWAS of the corresponding traits will be expected to discover more common variant-specific effects and fewer rare variant-specific effects. Third, as sample size increases, human phenome knockouts, i.e., complete loss of function by naturally occurring loss-of-function mutations, may be fruitfully studied by considering the phenotypic manifestations of lesser degree modulations of the genes through regulatory variations.

Several limitations need to be acknowledged. First, the current study used WES data to represent rare variants. This is a limitation given that the exome represents only 1–3% of the genome [7, 36]. In particular, intergenic signals from common variants would be challenging to match in WES-based studies. Follow-up studies with WGS data are needed to capture the rare variants in noncoding regions [36]. Second, the genes identified through MAGMA may not be causal. Indeed, empirical evidence suggests that only about one-third of the genes located nearest to the sentinel GWAS signals are potentially causal [6]. These two limitations imply that the convergence level is likely to be underestimated. Third, although we verified our main conclusion using independent data sources for the common and rare variant analyses, use of large-scale datasets such as *AllofUs* will further enhance the reliability of the finding. Fourth, we simplified the LD structure by picking one gene from each LD block. Future studies that model more complex LD patterns will further fine-tune our results. The latter two limitations may be addressed by future multi-ancestry studies. For example, as African populations have shorter LD blocks [41] (because of the larger effective population size of ancestral Africans and the greater time for recombination to reduce LD), the integration of genetic datasets in African populations may substantially improve the CORAC estimate by enhancing our understanding of causal genes from common and rare variants.

## Conclusions

We defined the COmmon variant and RAre variant Convergence (CORAC) signature for complex traits and found that the effective sample size considerably explained the signature. Thus, future WGS/GWAS will be expected to show increasing functional convergence of common and rare variant associations. Using both empirical data and simulations, we provide evidence that negative selection would not only explain a high degree of polygenicity for complex traits but also dampen the convergence level. Our framework provides a generalizable approach to rigorously investigate the level of concordance of effector genes across the allele frequency spectrum, informing future fine-mapping studies and uncovering the extent of heterogeneity of gene-level mechanisms.

### Supplementary Information

**Additional file 1: Fig. S1.** The convergence levels across different health domains. **Fig. S2.** Convergence (gwet_ac1) of common and rare genetic effects as a function of sample size. **Fig. S3.** Stratified QQ plots for rare variant-based genome-wide scan.

**Additional file 2: Table S1.** The CORAC value for each trait (common variant source: UK Biobank; rare variant source: UK Biobank). **Table S2.** The CORAC value for each trait (common variant source: GWAS ATLAS; rare variant source: UK Biobank).

## Data Availability

The genotype and phenotype data for the UK Biobank (https://www.ukbiobank.ac.uk/) samples were accessed under application number 102158 (Applicant PI: Dan Zhou). The GWAS and WES summary statistics which were generated by Neale lab are publicly accessible at http://www.nealelab.is/uk-biobank [19] and https://app.genebass.org/ [18], respectively. The GWAS summary statistics from the GWAS ATLAS (https://atlas.ctglab.nl/) was generated by Watanabe and colleagues [33]. The scripts are available from the Gamazon lab at Github (https://github.com/gamazonlab/CORAC).

## Abbreviations

CORAC: COmmon variant and RAre variant Convergence
GWAS: Genome-wide association study
WES: Whole-exome sequencing
WGS: Whole-genome sequencing
MAF: Minor allele frequency
LD: Linkage disequilibrium
PRS: Polygenic risk score
