# Supplementary material for: A phenome-wide scan reveals convergence of common and rare variant associations
Source: Genome Med. 2023 Nov 28;15:101. doi: 10.1186/s13073-023-01253-9 (PMC10683189; doi:10.1186/s13073-023-01253-9)
Supplement: Supplementary file 1 — Additional file 1: Fig. S1. The convergence levels across different health domains. Fig. S2. Convergence (gwet_ac1) of common and rare genetic effects as a function of sample size. Fig. S3. Stratified QQ plots for rare variant-based genome-wide scan. [file 13073_2023_1253_MOESM1_ESM.docx]

**A phenome-wide scan reveals convergence of common and rare variant associations**


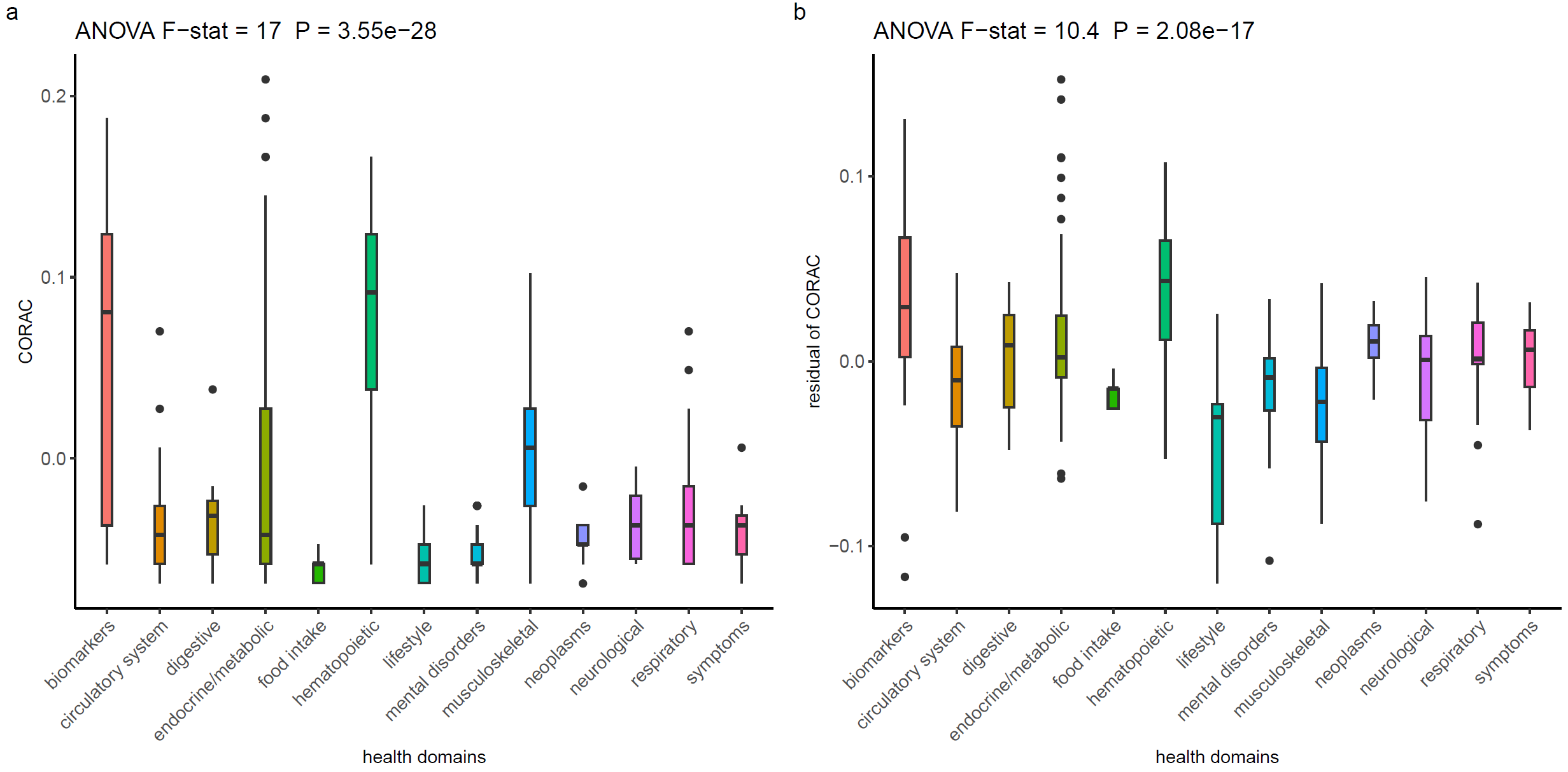


**Fig. S1. The convergence levels across different health domains.** We grouped the UK Biobank traits into health domains using the PheWAS categories. Analysis of Variance (ANOVA) was performed to compare the convergence level (CORAC) across the health domains, each with at least 5 traits. The boxplots show the CORAC levels before and after regressing out the effective sample size in panel a and b, respectively. The *F*-stat and *P*-value from the ANOVA is shown.


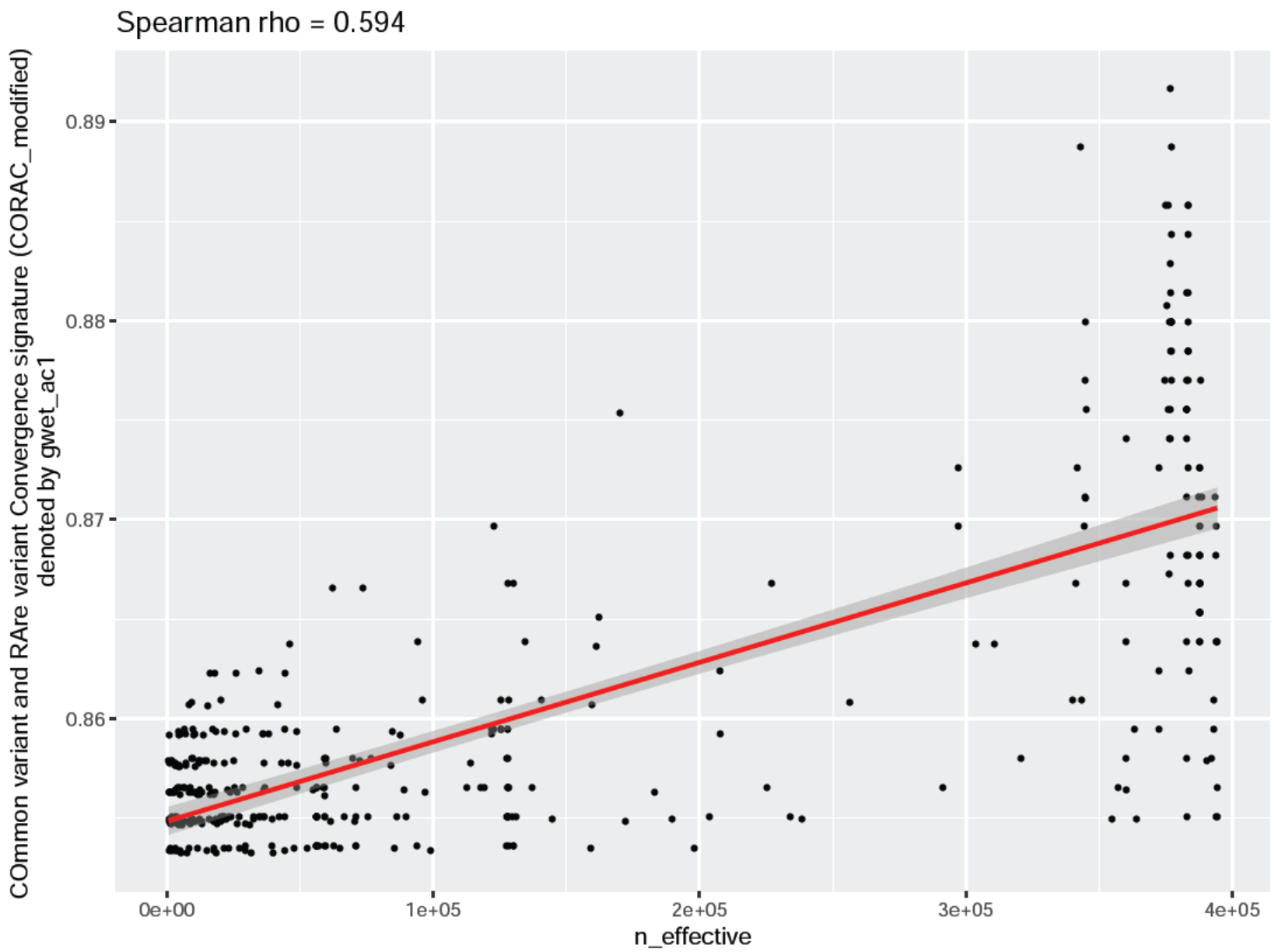


**Fig. S2. Convergence (gwet_ac1) of common and rare genetic effects as a function of sample size.** The CORAC_modified_ statistic (y-axis) is positively correlated with effective sample size (x-axis). The regression line and the 95% confidence bands are shown, allowing identification of traits with higher or lower convergence signature than expected given the effective sample size.


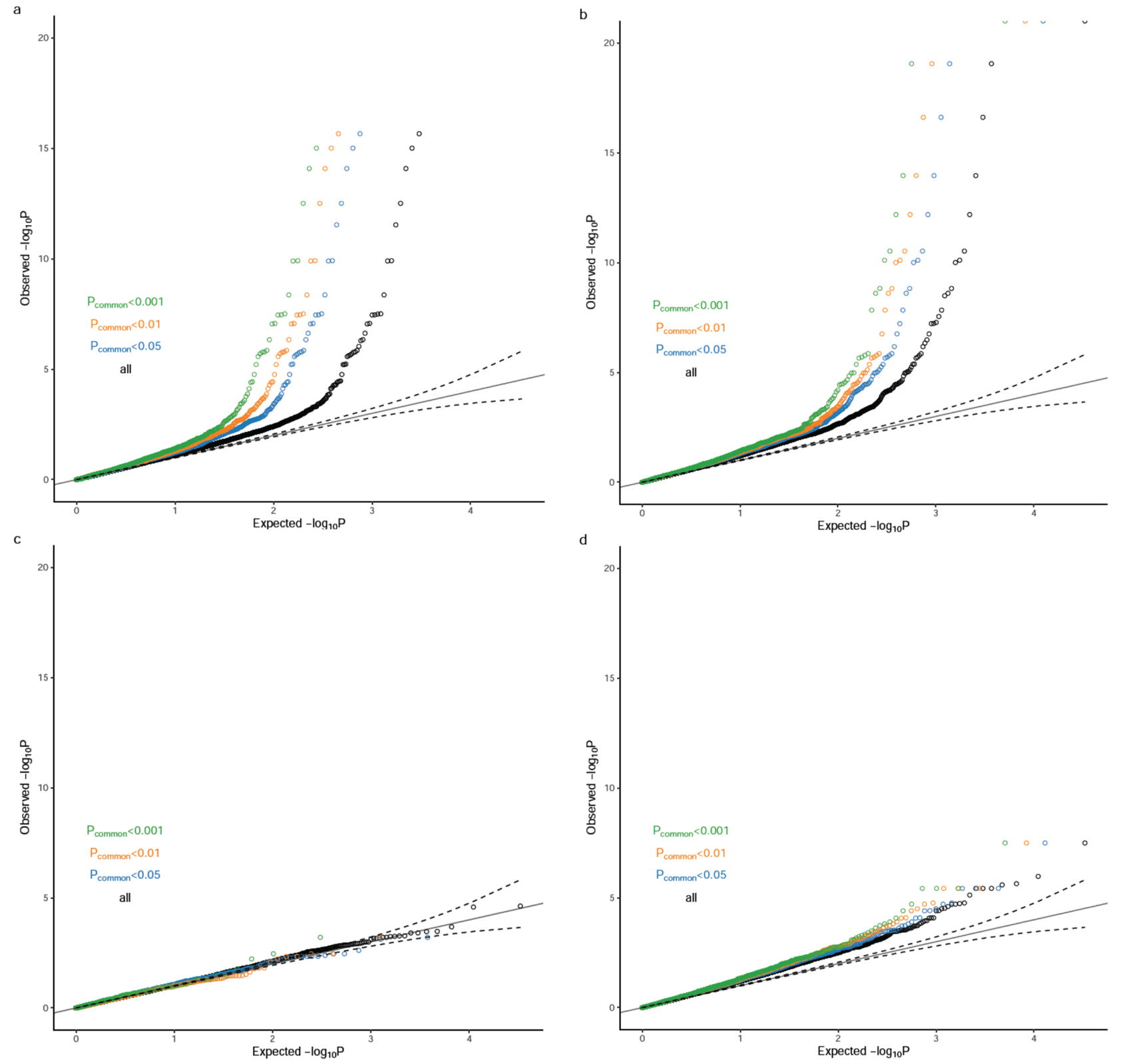


**Fig. S3. Stratified QQ plots for rare variant-based genome-wide scan.** We hypothesized that CORAC may be effective in restricting the search space for trait-associated genes, leading to enhanced association analysis. Stratified by the common variant-based gene-level p-value, the QQ plot of the rare variant-based gene-level results were generated for traits with high CORAC levels (**a** and **b** for cholesterol and cystatin C, respectively) and low CORAC levels (**c** and **d** for Townsend deprivation index at recruitment and body mass index, respectively).
